# Missing Screw as a Rare Complication of Anterior Cervical Instrumentation

**DOI:** 10.1155/2013/593905

**Published:** 2013-03-24

**Authors:** Yusuf Kurtuluş Duransoy, Mesut Mete, Baha Zengel, Mehmet Selçukı

**Affiliations:** ^1^Celal Bayar University, School of Medicine, Neurosurgery Department, Manisa, Turkey; ^2^Izmir Bozyaka Training and Research Hospital, General Surgery Department, Izmir, Turkey

## Abstract

Although anterior cervical arthrodesis is an effective procedure for the treatment of cervical disorders, the method has some complications. Here, we describe this rare complication of cervical instrumentation with a literature review. A 23-year-old male patient was operated for a C6-C7 dislocation. At postoperative month 10, he presented with hemoptysis and dysphagia. Cervical roentgenograms showed anterior migrations of one broken screw and a plate-locking screw at the C6 corpus. One screw was missing. We concluded that the missing screw had perforated the esophagus and had been eliminated spontaneously through the gastrointestinal tract. No screw should migrate. Even loose screws should be noted in follow-up X-ray studies. If such findings are detected, a second operation for revision should be considered as soon as possible to prevent potentially fatal complications.

## 1. Introduction

Anterior cervical spine surgery has been used in the treatment of cervical disc herniation, trauma, neoplasm, spondylosis, osteomyelitis, and ossification of the posterior longitudinal ligament [1, 2]. Although the method including anterior cervical decompression, fusion, and anterior stabilization is a well-established technique, it has some complications, including dysphagia [3], extrusion of screws and plates [4, 5], bone graft failure, cerebrospinal fluid leakage, recurrent laryngeal injuries [2], pharyngoesophageal injuries, prevertebral abscess, airway obstruction, mediastinitis, and carotid artery rupture [3]. Here, we present a case with a missing screw, which was discovered on follow-up radiological examination. We believe the screw was most probably spontaneously eliminated, with extrusion of the screw through the gastrointestinal tract following esophageal perforation.

## 2. Case Report

**Figure 1:**
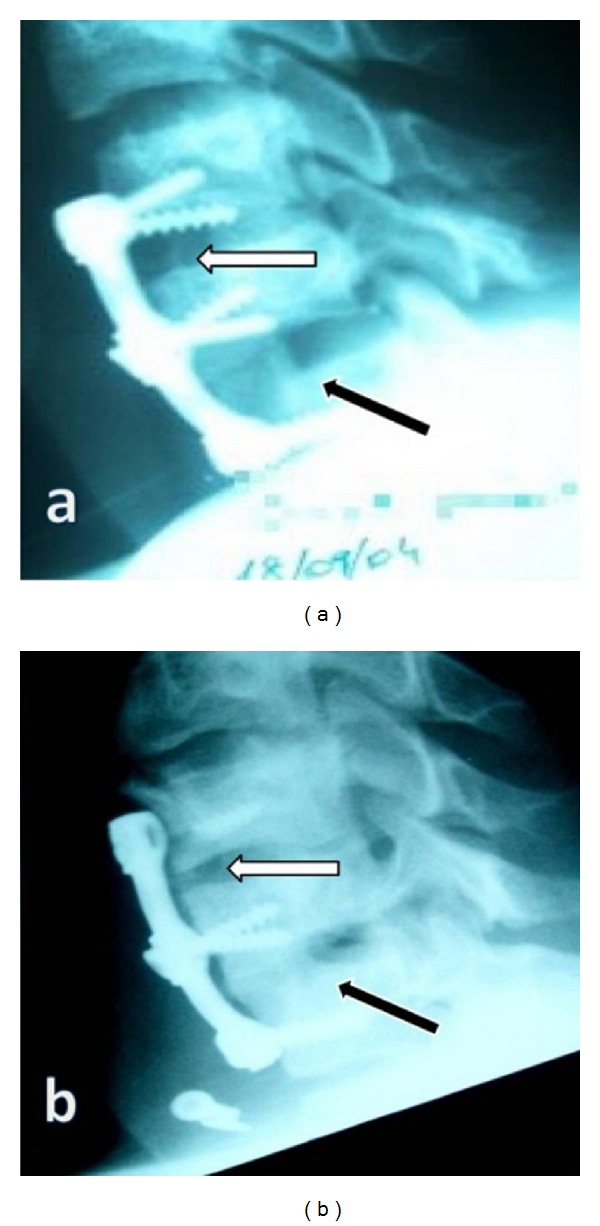
(a) Bone graft was seen at level C6-7 (black arrow), and there was no bone graft at C5-6 level (white arrow). (b) White arrow shows collapse at C5-6 level, and black arrow shows fusion by bone graft at C6-7 level.

A 23-year-old male patient was previously operated on for C6-C7 dislocation 10 months earlier in another institution. Surgical treatment included anterior discectomy and iliac crest graft placement at the C6-C7 level, untouched C5-6 level, and plate-screw fixation performed between the C5–C7 levels. Postoperative early roentgenogram after the previous operation showed error in operative design (Figure 1(a)). Late roentgenogram after 10 months from the previous operation showed collapse at C5-6 level (Figure 1(b)).

**Figure 2:**
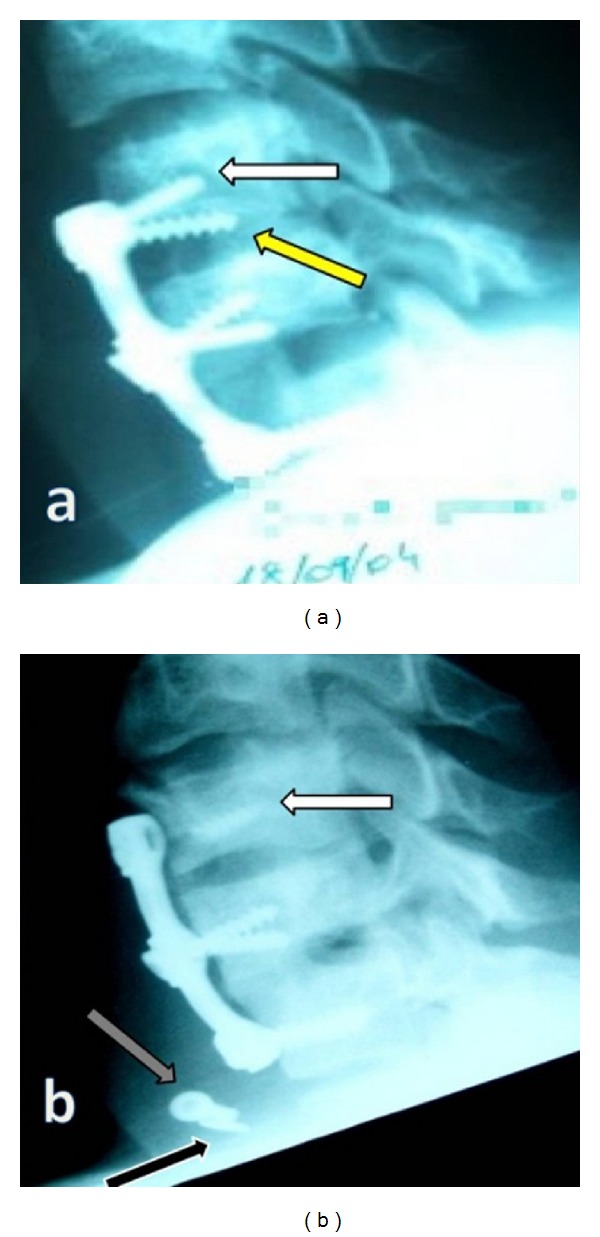
Cervical roentgenograms showed anterior migration of one broken screw and plate-locking screws at the C6 corpus. The yellow arrow shows the missing screw (a), black arrow indicates a migrated plate-locking screw, the white arrow shows distal part of the broken screw, and grey arrow shows the proximal part of the broken screw (b).

**Figure 3:**
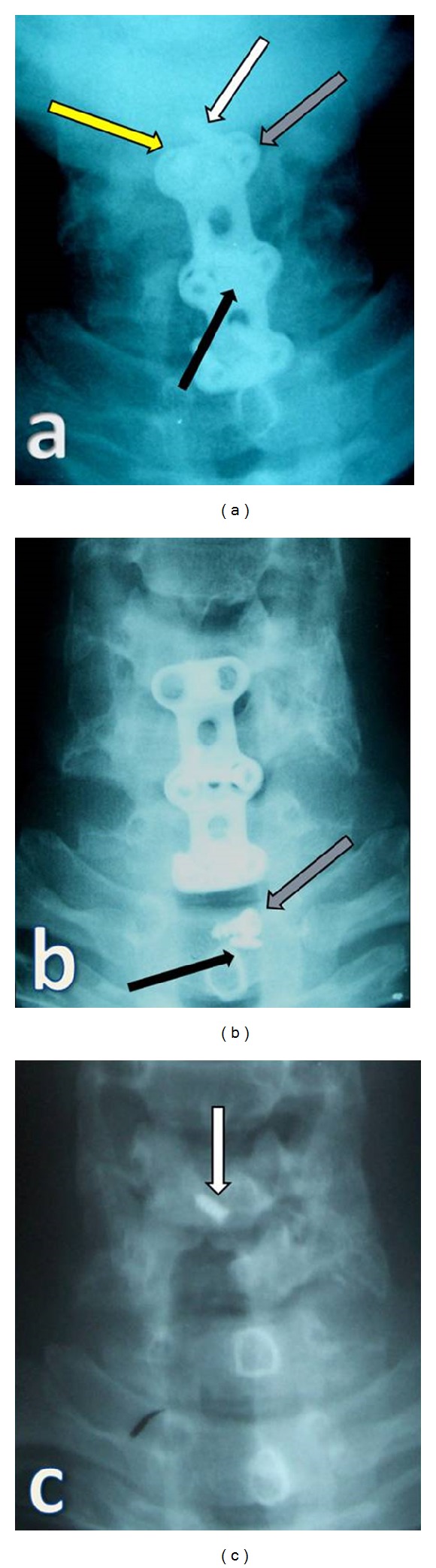
Yellow arrow shows missing screw, white arrow shows distal part of the broken screw, grey arrow shows proximal part of the broken screw, and black arrow indicates a migrated plate-locking screw (a), (b). After removal of all cervical instruments, distal part of the broken screw was still in C6 corpus (white arrow) (c).

At 10 months after the operation, he presented with hemoptysis and dysphagia. Cervical roentgenograms showed anterior migration of one broken screw and plate-locking screw at the C6 corpus. In Figures 2 and 3, the black arrow indicates a migrated plate-locking screw, the white arrow shows distal part of the broken screw, grey arrow shows the proximal part of the broken screw, and the yellow arrow shows missing screw.

Chest, abdominal, and pelvic roentgenograms failed to reveal the missing screw. During a second operation, the missing screw was confirmed, and we found the head portion of the broken screw and the plate-locking screw. We also noted the empty screw hole in the cervical plate. We did not see esophageal perforation. Cultures were negative. Because we determined that fusion at the C6-C7 level was complete, all instruments were removed. Esophagography was normal, and a fistula test with methylene blue was negative. As a result, we concluded that the missing screw had perforated the esophagus and had been eliminated spontaneously through the gastrointestinal tract.

## 3. Discussion

Anterior cervical arthrodesis and instrumentation is an effective procedure for the treatment of cervical disorders, including cervical disc herniation, trauma, neoplasm, spondylosis, osteomyelitis, and ossification of the posterior longitudinal ligament [1, 2]. Anterior cervical implants are chosen to improve the overall union rate and decrease the need for restrictive external immobilization [6]. Complications in anterior approaches decrease as the surgeon's experience increases. In a series of over 850 cases of anterior spine surgery, Cloward classified operative complications into three categories. These were soft tissue lesions, spinal cord or root injuries, and problems related to spinal stabilization [7]. Martínez-Lage et al. reported that graft dislodgement occurred in 6%–10% of patients, while Lowery and McDonough showed the incidence of instrumentation failure to be as high as 35% [8]. In addition, Lee et al. reported 2%–35% plate-related failure in anterior cervical fusions in their literature review [9]. The main predisposing factor to the development of screw and plate extrusion is suboptimal positioning of these screws [10]. Failure of the anterior plate screw system may cause serious complications, with possible damage to the esophagus, trachea, and vessels [2]. Daniel Riew et al. reported a case of plate rupture and graft migration causing airway impairment and resulting in the death of the patient [11].

There are some reports on the displacement of grafts and screws resulting in esophageal perforation. Pompili et al. reported a missing screw 2 years after anterior decompression from C4 to C7, and esophagoscopy was normal. They concluded that the screw had likely perforated the esophagus and was eliminated spontaneously through the gastrointestinal tract [2]. Chataigner et al. reported spontaneous elimination of an anterior fixation device through the gastrointestinal tract with a good outcome [12]. Wong et al. reported the case of a 56-year-old man with a missing screw after an anterior cervical decompression and fusion. The missing screw caused the late postoperative complication of upper airway obstruction as a result of a prevertebral abscess [5]. Martínez-Lage et al. described a 51-year-old man who underwent anterior cervical decompression and fusion. Six years later, cervical radiographs showed a missing screw, and esophagoscopy was normal [3]. Similar cases were also reported by Fujibayashi et al. and Cagli et al. They reported missing screws after anterior cervical decompression and fusion [6, 10]. Although it may be a fatal complication, few uneventful patients have been reported with esophagus perforation, and missing screws were found. Geyer and Foy reported a 76-year-old woman with oral extrusion of a missing locking plate 5 years after a C4-C5 decompression and fusion [4]. Lee et al. demonstrated endoscopic extrusion of a screw after anterior cervical disc fusion, which caused esophageal perforation [9]. Yee and Terry reported a patient with complete screw migration that perforated the esophagus. The missing screw was found in the lower gastrointestinal tract. During surgery, the surgeon found no evidence of esophageal perforation [13].  Table 1 shows previous reports about missing screws after an anterior cervical approach. Failure of anterior cervical instrumentation occurred in our patient because of no bone graft in C5-6 level which means error in operative design (Figures 1(a) and 1(b)).

Esophageal injuries can be divided into intraoperative and delayed postoperative complications. Intraoperative complications can occur at the time of surgery or during the early postoperative phase, such as a decubitus wound [1]. Because posterior esophageal mucosa is thin and covered only by fascia at this point, esophageal perforation is seen more often at the C5-C6 level during anterior cervical spine surgery [14]. Complications due to esophageal perforation range from local infections to mediastinitis and death to spontaneous recovery [1, 4, 14]. If treatment is delayed, the mortality for all causes of esophageal perforation is 20%–50% [2]. Spontaneous recovery from esophageal perforation was described by Pompili et al. They identified no esophageal scarring in their patients. This may result from the small diameter of the screw and the slow migration process. Authors reported that this process must have been enabled in the healing of tissues and the repair of the defect, plane by plane, from the external to the internal mucosa [2]. The patients presented with neck pain, dysphagia, weight loss, fever, difficulty swallowing, and subcutaneous cervical emphysema [10].

Management of esophageal perforation depends on the time of the laceration. Conservative treatment consisting of intravenous antibiotics, elimination of oral intake, and parenteral nutrition can be preferred for minimal defects [6]. If the perforation is discovered during surgery, repair by sutures or a flap with sternocleidomastoid muscle should be performed. When perforation is detected in the first days following the operation, surgical closure of the lesion, drainage, and irrigation of the operative area should be performed [1]. In delayed esophageal perforation, all anterior cervical instruments are removed if patients have evidence of fusion. Otherwise, posterior cervical instrumentation should be applied after removing of all instruments [15]. We removed all cervical instruments because of fusion at C6-7 level.

Although loosening of anterior cervical instruments is not rare, the number of patients reported with a missing screw following loosening is small [2, 3, 5, 6, 10]. To prevent this complication, surgeons should be sure about the optimal positioning of instruments during surgery (a locking screw should be used, and screws should not be in disc spaces). Contact between the plate and underlying bone must be sufficient, and greater attention is required for sufficient fusion. 

## 4. Conclusions

1. Although anterior instrumentation is safe for use during surgery to treat cervical spine disorders, this approach has some complications because of the complex anatomy of the anterior neck region.
2. Failure of an anterior instrumentation system may cause rare serious complications, with possible damage to the esophagus, trachea, and vessels. For this reason, patients who undergo anterior cervical instrumentation should be followedup for a period of time to detect whether there may be a failure of the anterior plate screw system.
3. No screw should migrate. Even loose screws should be noted in follow-up X-ray studies. If such findings are detected, a second operation for revision should be considered as soon as possible to prevent potentially fatal complications.

## Figures and Tables

**Table 1:** Missing screws and locking plates after an anterior cervical approach in the literature (Pubmed).

Author	Symptoms	F/T	Result	Fusion level	Missing instrumentation
Fujibayashi et al., 2000 [6]	High fever, meningitis, dysphagia	15 m	Died	C4–T1	1 screw
Pompili et al., 2002 [2]	Asymptomatic	6 m	Recovered	C4–C7	1 screw
Wong et al., 2005 [5]	Airway obstruction, cervical abscess	4 y	Recovered	C3–C6	1 screw
Martínez-Lage et al., 2007 [3]	Dysphagia, fever, neck swelling	6 y	Recovered	C5–C7	1 screw
Cagli et al., 2009 [10]	Dysphagia	7 y	Recovered	C4–C7	1 screw

F/T: follow-up time, m: month, y: year.
